# Rho GTPase Expression in Human Myeloid Cells

**DOI:** 10.1371/journal.pone.0042563

**Published:** 2012-08-16

**Authors:** Suzanne F. G. van Helden, Eloise C. Anthony, Rob Dee, Peter L. Hordijk

**Affiliations:** 1 Department of Molecular Cell Biology, Sanquin Research, Amsterdam, The Netherlands; 2 Landsteiner Laboratory, Academic Medical Centre, University of Amsterdam, Amsterdam, The Netherlands; 3 Department of Immunocytology, Sanquin Diagnostics, Amsterdam, The Netherlands; Center of Ophtalmology, Germany

## Abstract

Myeloid cells are critical for innate immunity and the initiation of adaptive immunity. Strict regulation of the adhesive and migratory behavior is essential for proper functioning of these cells. Rho GTPases are important regulators of adhesion and migration; however, it is unknown which Rho GTPases are expressed in different myeloid cells. Here, we use a qPCR-based approach to investigate Rho GTPase expression in myeloid cells.

We found that the mRNAs encoding Cdc42, RhoQ, Rac1, Rac2, RhoA and RhoC are the most abundant. In addition, RhoG, RhoB, RhoF and RhoV are expressed at low levels or only in specific cell types. More differentiated cells along the monocyte-lineage display lower levels of Cdc42 and RhoV, while RhoC mRNA is more abundant. In addition, the Rho GTPase expression profile changes during dendritic cell maturation with Rac1 being upregulated and Rac2 downregulated. Finally, GM-CSF stimulation, during macrophage and osteoclast differentiation, leads to high expression of Rac2, while M-CSF induces high levels of RhoA, showing that these cytokines induce a distinct pattern. Our data uncover cell type specific modulation of the Rho GTPase expression profile in hematopoietic stem cells and in more differentiated cells of the myeloid lineage.

## Introduction

Within the immune system many different cell types carry out specific roles to ensure proper immunity, both innate as well as adaptive. Cells of myeloid origin mediate innate immune responses but are also essential for the initiation of adaptive immunity. The myeloid lineage includes neutrophils that form the first line of defense, as well as monocytes, macrophages and dendritic cells (DCs), which are crucial for initiation of T cell responses [1]. A common feature of these cells is their ability to migrate, which is a tightly controlled process.

Rho GTPases are important regulators of the actin cytoskeleton and thereby control the adhesive and migratory behavior of cells. Rho GTPases cycle between an inactive, GDP-bound form and an active, GTP-bound form. The guanine nucleotide-exchange factors (GEFs) regulate the activation of Rho GTPases by promoting the exchange of GDP for GTP, while GTPase-activating proteins (GAPs) promote the intrinsic GTPase activity and thus the transition back to the GDP-bound state [2]–[3]. GDP-bound Rho GTPases are sequestered by Rho guanine nucleotide dissociation inhibitor (RhoGDI) [4], which serves as a molecular chaperone and regulator to protect the cell from aberrant GTPase activation. The balanced action of GEFs and GAPs is crucial for proper functioning of Rho GTPases and controls the timing and localization of Rho GTPase activity. The GTP-bound forms of the Rho GTPases transduce signals by binding to effector proteins, inducing a conformational change or altered localization, which is in turn required to transmit signals further downstream. In addition, Rho GTPases are regulated at the level of stability and expression.

The Rho GTPases can be divided in classical and atypical (Table 1). The classical Rho GTPases cycle between the active and inactive state as described above. The atypical Rho GTPases, either through mutations in the GTP-domain or through elevated nucleotide exchange ability, are constitutively GTP bound [5]–[11]. Therefore, regulation by GEFs and GAPs may be less important for atypical Rho GTPases and regulation on expression level or by modifications is more prominent. The family of Rho GTPases contains 20 members and their splice variants that can be divided into various subfamilies, i.e. Cdc42, Rac, Rho, RhoF, RhoU, Rnd, and RhoBTB and RhoH (Table 1) [12]. Within these subfamilies, members often share effectors and can be regulated by the same GEFs and GAPs. Specificity of Rho GTPase signaling is determined by specific subcellular localization as well as cell-type specific expression of the different GTPases and their regulators [13].

**Table 1:** The family of Rho GTPases.

Subfamily	Rho GTPase	GeneID	Type
Cdc42-like	Cdc42	998	Classical
	RhoJ (TCL)	57381	Classical
	RhoQ (TC10)	23433	Classical
Rac-like	Rac1	5879	Classical
	Rac2	5880	Classical
	Rac3	5881	Classical
	RhoG	391	Classical
Rho-like	RhoA	387	Classical
	RhoB	388	Classical
	RhoC	389	Classical
RhoF	RhoD	29984	Classical
	RhoF (Rif)	54509	Classical
RhoH	RhoH (TTF)	399	Atypical
RhoU	RhoU (Wrch1)	58480	Atypical
	RhoV (Wrch2/Chp)	171177	Atypical
RhoBTB	RhoBTB1	9886	Atypical
	RhoBTB2	23221	Atypical
Rnd	Rnd1	27289	Atypical
	Rnd2	8153	Atypical
	Rnd3 (RhoE)	390	Atypical

Rho GTPases can be classified as classical or atypical. Classical Rho GTPases cycle between the GDP- and GTP-bound form, while atypical Rho GTPases are almost always in the active form. GeneIDs were derived from http://www.ncbi.nlm.nih.gov/.

Human hematopoietic progenitor cells, marked by the expression of CD34, give rise to the different progenitors and cells of the lymphoid and myeloid lineage [14]. Within the myeloid lineage there are two different progenitors, i.e. the megakaryocyte-erythrocyte progenitor (MEP) and the granulocyte-monocyte progenitor (GMP). The MEP ultimately gives rise to platelets and erythrocytes. The GMP gives rise to the different granulocytes, i.e. neutrophils, eosinophils and basophils, as well as to monocytes, DCs, macrophages, mast cells and osteoclasts [14]–[16]. Here, we focus on these GMP-derived cells. The expression of the different Rho GTPases in these cells is largely unknown. Therefore, we determined the cell type specific expression of the 20 family members and their splice variants in different types of myeloid cells by a qPCR based approach.

## Results

### Isolation and culture of different human myeloid cells

**Figure 1:**
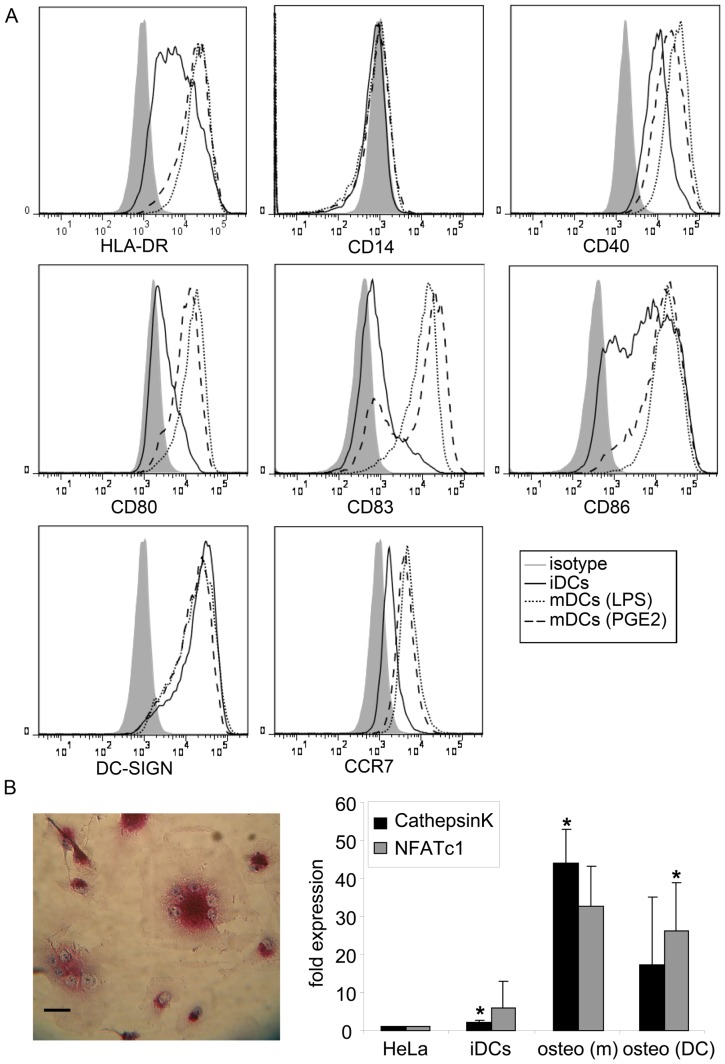
Confirmation of cell differentiation. (A) Flow cytometric analysis of immature and mature DCs. Filled graphs represent isotype controls (isotype), solid lines represent immature DCs (iDCs), dotted lines represent LPS-matured DCs (mDCs (LPS)) and dashed lines represent PGE_2_-matured DCs (mDCs (PGE2)). Surface expression of the monocyte marker CD14 is low. HLA-DR and DC-SIGN are highly expressed. The costimulatory molecules CD40, CD80 and CD86 are upregulated during differentiation. The maturation markers CD83 and CCR7 are expressed upon maturation. (B) Corfirmation of osteoclast generation. The left panel shows a TRACP staining of osteoclasts. Images were obtained using a Zeiss LSM 510-meta microscope with a Plan-Apochromatic 63× 1.4 NA oil immersion objective (Carl Zeiss, Jena, Germany) in combination with a camera (Pentax Europe GmbH, Germany). The magenta staining shows the presence of tartrate-resistant phosphate in the osteoclasts generated from monocytes. In addition, the multiple nuclei can be seen in blue. Representative image is shown. Scale bar; 20 µm. In the bar graph, the expression of the osteoclasts markers cathepsinK and NFATc1 is depicted as compared to expression in HeLa (fold expression). The expression of cathepsinK and NFATc1 was analyzed (n = 3) in HeLa, immature DCs (iDCs), monocyte-derived osteoclasts (osteo (m)) and DC-derived osteoclasts (osteo (DC)). The expression of these markers is upregulated in osteoclasts. * indicates significant difference from HeLa; *p*<0.05. Together, this shows the successful generation of osteoclasts.

To compare the Rho GTPase expression profile in different types of myeloid cells, we isolated the various cell types, prior to qPCR based analysis of the different Rho GTPase transcripts. We isolated monocytes by adherence to plastic or by CD14^+^-Macs isolation and differentiated these further to macrophages, dendritic cells (DCs) and osteoclasts. The monocytes obtained with CD14^+^-Macs isolation were 93% pure as determined by CD14 surface expression (data not shown). Immature DCs displayed high surface expression levels of HLA-DR, CD86, CD40 and DC-SIGN, intermediate levels of CD80 and lacked expression of CD14, showing that these DCs had an immature phenotype (Figure 1A). Upon maturation with LPS or PGE_2_ for 24 hours the surface expression of costimulatory molecules, i.e. CD40, CD80, CD86, CD83, and the chemokine receptor CCR7 were upregulated, confirming that these DCs had matured (Figure 1A). The upregulation of these markers is somewhat more in LPS-matured DCs than in PGE_2_-matured DCs, suggesting that LPS-induced maturation might be more robust. To confirm the generation of osteoclasts, we stained for Tartrate-Resistant ACid Phosphatase (TRACP). TRACP-positive cells and multinucleated cells were observed in cultures derived from monocytes or DCs (Figure 1B left panel and data not shown). In addition, the expression of the osteoclast markers CathepsinK and NFATc1 [17]–[18] was analyzed by qPCR in osteoclasts and immature DCs and compared to expression in HeLa cells (Figure 1B right panel). The expression of cathepsinK was 2.1 times higher and 5.9 times higher for NFATc1 in immature DCs compared to HeLa (Figure 1B right panel). So the expression of these markers is already higher in immature DCs than in HeLa. The expression of cathepsinK was 44.1 times higher in osteoclasts derived from monocytes and 17.4 times higher in osteoclasts derived form DCs compared to HeLa (Figure 1B right panel). For NFATc1 this is 32.7 and 26.3 times, respectively (Figure 1B right panel). The expression of cathepsinK and NFATc1 is much higher in both types of osteoclasts than in HeLa or immature DCs. Together, these data confirm the successful generation of osteoclasts.

**Figure 2:**
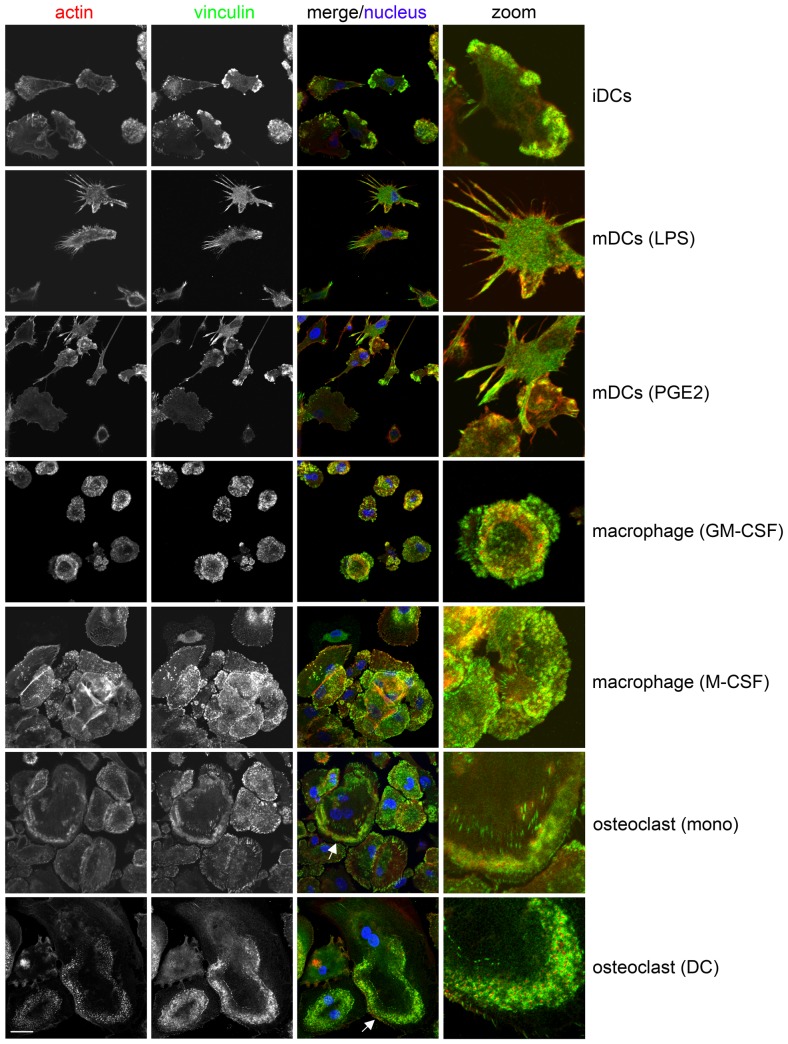
Morphology of differentiated myeloid cells on fibronectin. The cells are seeded on fibronectin-coated coverslips and stained for vinculin (green, second column). Phalloidin Texas Red (red, first column) is used to detect F-actin and Hoechst33258 is used to visualize nuclei (blue, third column). Images were obtained by confocal microscopy using a Zeiss LSM 510-meta microscope with a Plan-Apochromatic 63× 1.4 NA oil immersion objective (Carl Zeiss, Jena, Germany) and analyzed using Zen software (Carl Zeiss). The third column shows the merged image and the fourth column shows a zoom of a part of the merge image. Podosomes can be seen as actin dots surrounded by vinculin rings. In the osteoclasts podosome rings (arrows) can be observed. iDCs; immature DCs, mDCs (LPS); LPS-matured DCs, mDCs (PGE2); PGE_2_-matured DCs. Representative images are shown. Scale bar; 20 µm.

In addition, the phenotype of the different types of cells was assessed by confocal microscopy, following immunofluorescent staining for vinculin and F-actin. Immature DCs adhered to and spread on fibronectin (Figure 2, upper panel). Most of the immature DCs were polarized and formed podosomes, specialized adhesion structures in myeloid cells capable of matrix remodeling [19]–[20], close behind the leading edge. Mature DCs, generated using LPS or PGE_2_, were weakly adherent, much less spread and (almost) devoid of podosomes (Figure 2). More podosomes remained in the PGE_2_-matured DC and the LPS-matured DCs displayed the typical dendrite-like extensions which these cells are named after. Macrophages generated with GM-CSF or M-CSF were adherent and spread on fibronectin (Figure 2). The majority of these cells were not polarized and podosomes could be observed distributed over the entire ventral surface of these cells. The osteoclasts, derived from monocytes or DCs were adherent, well spread cells that formed podosomes (Figure 2). In these osteoclasts, podosomes were organized into podosome rings and belts. Podosome rings are transient structures that mature into podosome belts in osteoclasts. Podosome belts are thought to give rise to the sealing zone, which is only observed in actively resorbing osteoclasts on bone [21].

### General expression pattern of Rho GTPases is similar in different cell types

**Figure 3:**
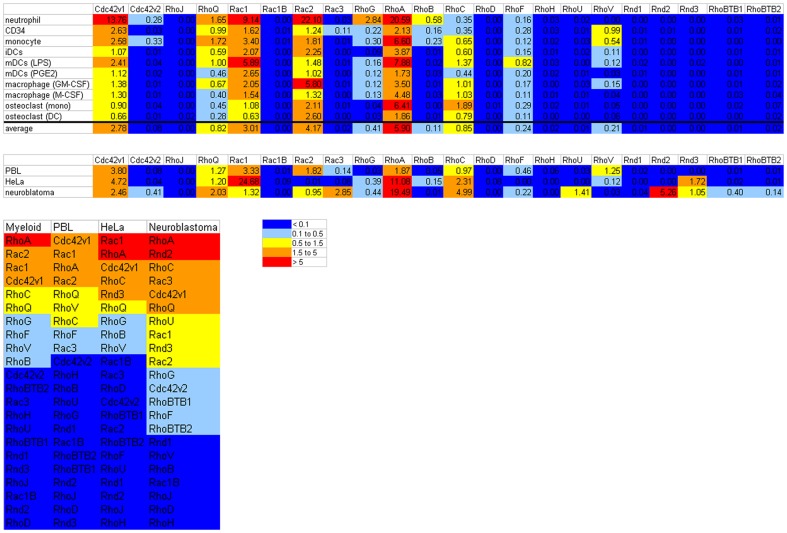
General expression pattern of Rho GTPases in myeloid cells. The mRNA expression of the Rho GTPases is depicted as the average 2^−ΔΔCt^ value per Rho GTPases for the individual cell types and averaged for myeloid cells (upper panel) and for the control cell types, i.e. PBLs, HeLa and neuroblastoma cells (second panel). CD34; CD34^+^ cells, iDCs; immature DCs, mDCs (LPS); LPS-matured DCs, mDCs (PGE2); PGE_2_-matured DCs. Expression of the Rho GTPases in each cell type was determined in 2 or 3 different donors or donormixes (see Materials and Methods). The colors mark the expression level. The lower panel shows a ranking of the Rho GTPase expression in myeloid cells and in the control cell types.

The expression of the different Rho GTPases at the mRNA level was determined in CD34^+^ cells, neutrophils, monocytes, immature and mature DCs, macrophages (generated with GM-CSF or M-CSF) and osteoclasts (generated from monocytes or from DCs). In figure 3 the expression of mRNA for each Rho GTPase is depicted as the average of the 2^−ΔΔCt^ values of the individual data points in the different cell types as well as the average expression in all the myeloid cells (also see Figure S1). There is a general pattern of Rho GTPases that are expressed at the mRNA level in myeloid cells and of GTPases that are not expressed or at a very low level (Figure 3). The 2^−ΔΔCt^ values of RhoJ, the Rac1 splice variant Rac1B, RhoD, RhoH, RhoU, Rnd1, Rnd2, Rnd3 and RhoBTB1 are below 0.1 in all myeloid cell types analyzed; therefore these Rho GTPases are probably less important in myeloid cells. Some of these Rho GTPases are well expressed in the cell types that we used as controls for the PCR approach, i.e. Peripheral Blood Lymphocytes (PBLs), HeLa and neuroblastoma cells and could have important functions there (Figure 3 and Figure S1). For example RhoU, RhoBTB1 and especially Rnd2 are expressed in neuroblastoma, while Rnd3 is expressed in HeLa and neuroblastoma. The hematopoietic RhoH is expressed in PBLs and the low expression of RhoH in myeloid cells suggests that this Rho GTPase has primarily a lymphoid, rather than myeloid, expression as was also previously suggested [22]–[23]. Although 2^−ΔΔCt^ values above 0.1 are measured for Rac3 and RhoBTB2 this is only in a few myeloid cell types and these values are below 0.2, therefore these Rho GTPases might be important in these cells but probably not in general for the myeloid lineage. For some Rho GTPases low levels of mRNA were detected (2^−ΔΔCt^ values below 1), i.e. Cdc42 splice variant 2, RhoG and RhoB. RhoG is only prominently expressed in neutrophils (which causes its relatively high ranking in myeloid cells in Figure 3, also see Figure S2). RhoB is expressed mainly by CD34^+^ cells, neutrophils and monocytes (Figure S2). The most prominently expressed Rho GTPases mRNAs in these myeloid cells from the granulocyte-monocyte lineage are Cdc42 splice variant 1, RhoQ, Rac1, Rac2, RhoA, RhoC, RhoF and RhoV (Figure 3 and Figure S2). For these Rho GTPases there are differences between the expression patterns in different cell types. For example, in CD34^+^ cells the Cdc42 splice variant 1 is the most abundant Rho GTPase, whereas this is Rac1 in PGE_2_-matured DCs, Rac2 in neutrophils and RhoA in monocytes.

**Figure 4:**
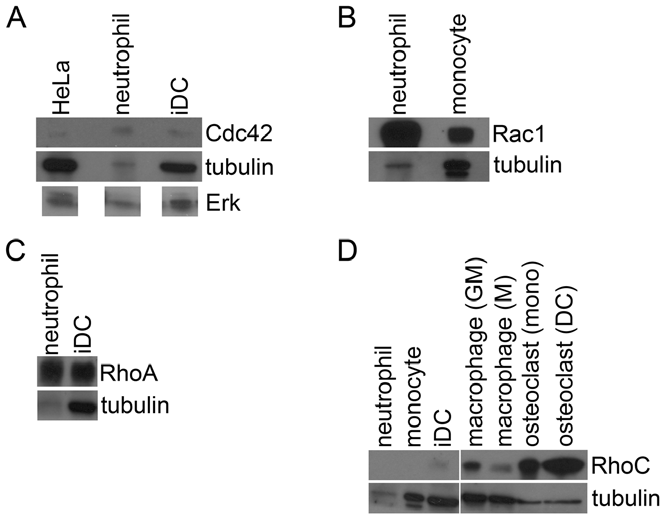
Expression of Rho GTPases on protein level. Western blots showing the protein levels of Cdc42, Rac1, RhoA and RhoC compared to tubulin. The levels of tubulin are comparable except for neutrophils where there is less protein loaded. (A) Protein expression of Cdc42. The expression of Cdc42 is compared to tubulin and total ERK. The levels of ERK are comparable to the tubulin levels. Depicted ERK bands were derived from one and the same western blot. Cdc42 protein expression is higher in neutrophils than in HeLa or immature DCs (iDC). (B) Protein levels of Rac1. Rac1 expression is higher in neutrophils than in monocytes. (C) Protein expression of RhoA. RhoA expression is higher in neutrophils than in immature DCs (iDC). (D) Protein levels of RhoC. RhoC expression is low in neutrophils (although the tubulin level is also low) and monocytes. Some expression is observed in immature DCs (iDC), while prominent expression is observed in macrophages differentiated with GM-CSF (GM) or M-CSF (M) and in osteoclasts derived from monocytes (mono) or DC (DC).

To compare the mRNA levels to protein levels, we investigated the protein levels of some Rho GTPases, i.e. Cdc42, Rac1, RhoA and RhoC, as proof of principle and used tubulin and ERK as a loading control (Figure 4). The expression of Cdc42 is higher in neutrophils than in HeLa or immature DCs, especially compared to the tubulin or ERK level, which is lower for the neutrophils (Figure 4A). Rac1 is more prominently expressed in neutrophils than in monocytes (Figure 4B) and the expression of RhoA is higher in neutrophils than in immature DCs (Figure 4C). The levels of RhoC are low in neutrophils and monocytes, somewhat higher in immature DCs and RhoC is prominently expressed on protein level in macrophages and osteoclasts (Figure 4D). These results show that in these examples the mRNA levels correlate well with the protein levels of these Rho GTPases.

### Expression of Rho GTPases in CD34^+^ cells, neutrophils and monocytes

**Figure 5:**
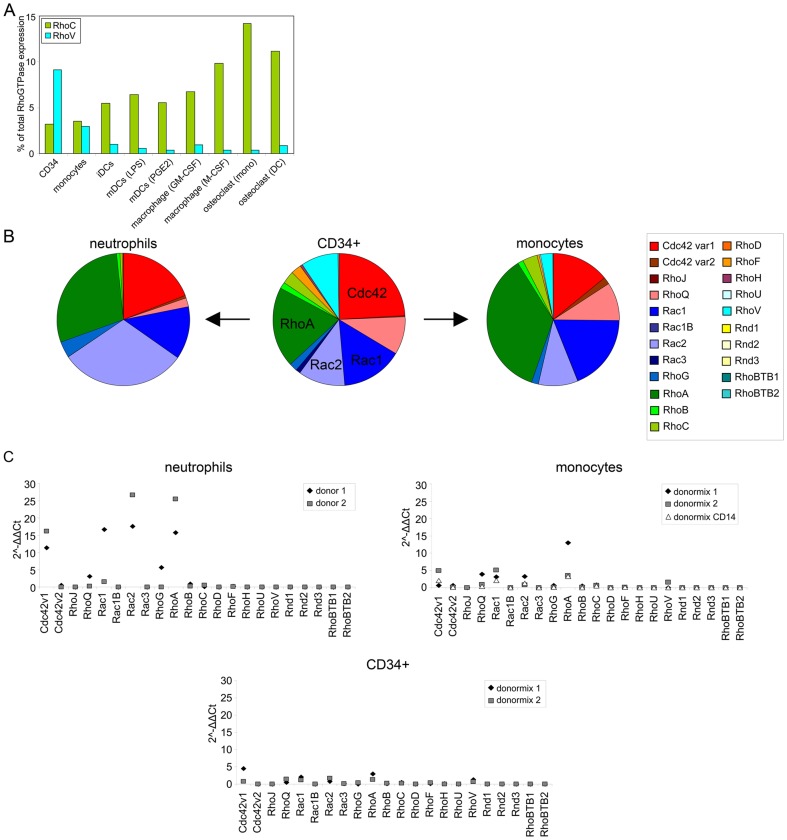
Rho GTPase expression compared in progenitor cells and differentiated myeloid cells. (A) Expression pattern of RhoC and RhoV in the monocyte-lineage. The expression of RhoC and RhoV are depicted as a percentage of total Rho GTPase expression. The expression of RhoC is lowest in CD34^+^ cells, low in monocytes and increases during differentiation of monocytes, while RhoV displays an inverse expression pattern. CD34; CD34^+^ cells, iDCs; immature DCs, mDCs (LPS); LPS-matured DCs, mDCs (PGE2); PGE_2_-matured DCs. (B) Rho GTPase expression in CD34^+^ cells, neutrophils and monocytes. The percentage of total Rho GTPase expression is depicted for each cell type in a pie chart. Rho GTPase subfamilies and individual Rho GTPases are color coded (for example Rho subfamily is green and RhoA is dark green). (C) The 2^−ΔΔCt^ values of the Rho GTPases in CD34^+^ cells, neutrophils and monocytes. The 2^−ΔΔCt^ values of the individual data points for each cell type are depicted. Donormix 1 and 2 are derived from 9 and 3 donors, resp. Donormix CD14 is derived from the same donors as donormix 2, but monocytes were obtained by elutriation followed by CD14 MACS isolation.

Human hematopoietic stem and progenitor cells express CD34 and give rise to different cell populations in the hematopoietic system, including important myeloid cells such as neutrophils or monocytes [14]. Monocytes can be further differentiated (*in vitro*) to generate macrophages, DCs and osteoclasts. During differentiation of cells along the monocyte-lineage, i.e. comparing CD34^+^ cells, monocytes and the different cell types derived from monocytes, the expression of RhoC markedly increased while the expression of RhoV decreased (Figure 5A and Figure S2). This suggests a cell-type specific regulation at the RNA level of these GTPases during differentiation.

Neutrophils and monocytes are two myeloid cell types that can be derived from CD34^+^ cells. To see if the Rho GTPase expression differs between the progenitor cells and these cells or between neutrophils and monocytes, we compared the expression pattern in CD34^+^ cells, neutrophils and monocytes either by looking at the percentage of total Rho GTPases expression in a cell type (Figure 5B) or by comparing the individual 2^−ΔΔCt^ values (Figure 5C). In CD34^+^ cells, Cdc42 is the most prominent Rho GTPase, while in neutrophils this is Rac2 (with RhoA being also very abundant) and in monocytes RhoA (Figure 5B). This already shows that the balance between the Rho GTPases is different between progenitor and differentiated cells and among differentiated cells. The 2^−ΔΔCt^ values, especially for Cdc42 splice variant 1, Rac1, Rac2 and RhoA, are very high in neutrophils (Figure 5C). The levels in monocytes and CD34^+^ cells are much lower, with the levels in monocytes being the higher of the two (Figure 5C). The level of Cdc42 splice variant 1 is similar in CD34^+^ cells and monocytes while the levels of RhoQ, Rac1, Rac2, RhoA and RhoC are higher in monocytes. Although the trend is that the expression of Rho GTPases is higher in the more differentiated neutrophils and monocytes, a few Rho GTPases display an alternative pattern, i.e. the expression of RhoF and RhoV is higher in CD34^+^ cells (Figure 5A and C).

### Expression during DC maturation

**Figure 6:**
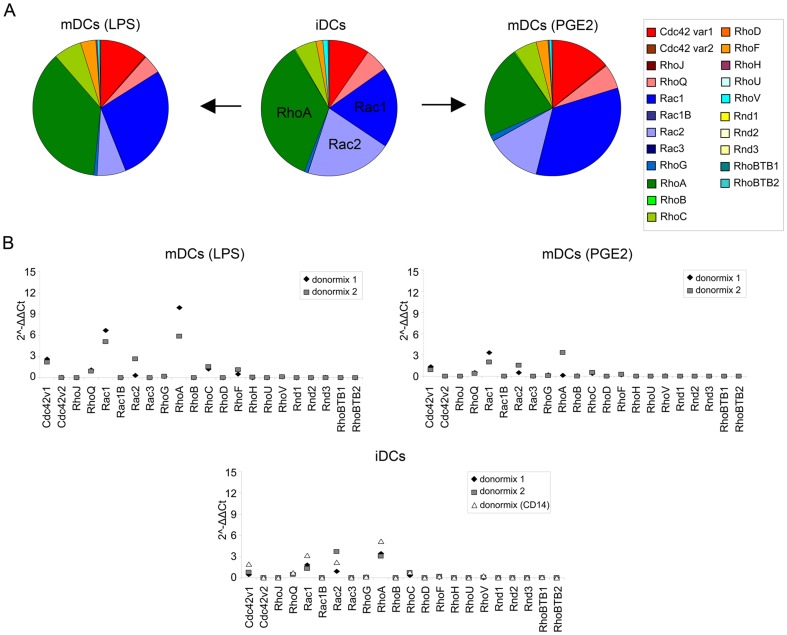
Rho GTPase expression during DC maturation. (A) Rho GTPase expression in DCs during maturation with LPS or PGE_2_. The percentage of total Rho GTPase expression is depicted for each DC in a pie chart. Rho GTPase subfamilies and individual Rho GTPases are color coded. iDCs; immature DCs, mDCs (LPS); LPS-matured DCs, mDCs (PGE2); PGE_2_-matured DCs. (B) The 2^−ΔΔCt^ values of the Rho GTPases in immature and mature DCs. The 2^−ΔΔCt^ values of the individual data points for each cell type are depicted. Donormix 1 and 2 are derived from 9 and 3 donors, resp. Donormix CD14 is derived from the same donors as donormix 2, but monocytes were obtained by elutriation followed by CD14 MACS isolation and differentiated to DCs. iDCs; immature DCs, mDCs (LPS); LPS-matured DCs, mDCs (PGE2); PGE_2_-matured DCs.

For proper induction of adaptive immunity it is critical that DCs undergo maturation during which molecules that are important for T cell stimulation are upregulated. Since this maturation is accompanied by a dramatic change in adhesive and migratory properties, we determined whether the expression of the Rho GTPases changes during maturation. Immature DCs were compared to DCs that were matured with LPS or with PGE_2_. In immature DCs and LPS-matured DCs RhoA is the most abundant Rho GTPase mRNA, while in PGE_2_-matured DCs this is Rac1 (Figure 6A). The expression pattern of the Rho GTPases is similar between immature and mature DCs. However, during maturation the levels of Cdc42 splice variant 1, RhoQ, Rac1, RhoG and RhoF increase, while the levels of Rac2 decrease during maturation (Figure 6B). These data suggest that balance between Rac1 and Rac2 changes during DC-maturation. These findings show that DCs modify the expression of a selection of Rho GTPases at the RNA level during the process of maturation.

Furthermore, the idea that LPS has induced more potent maturation, as determined by marker expression and podosome dissolution, is supported by the pattern of Rho GTPase mRNA levels, since the pattern in PGE_2_-matured DCs seems mostly intermediate between immature and LPS-matured DCs. There are two exceptions; RhoA and RhoC. The mRNA levels of both these Rho GTPases decreases upon maturation with PGE_2_, while they increase in response to LPS (Figure 6B), suggesting that different maturation stimuli can to some extent generate different mature DCs.

### GM-CSF and M-CSF induce expression of different Rho GTPases

**Figure 7:**
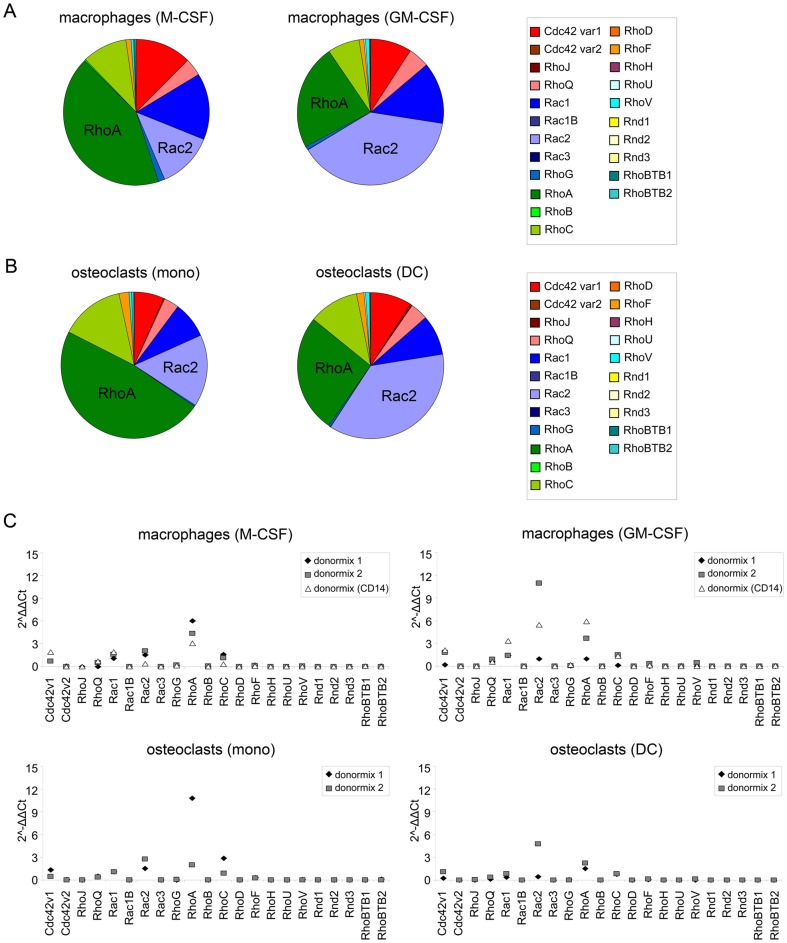
Rho GTPase expression in macrophages and osteoclasts. (A) Rho GTPase expression in macrophages generated with M-CSF or GM-CSF. The percentage of total Rho GTPase expression is depicted for each macrophage type in a pie chart. Rho GTPase subfamilies and individual Rho GTPases are color coded. (B) Rho GTPase expression in osteoclasts generated from monocytes or DCs. The percentage of total Rho GTPase expression is depicted for each osteoclast type in a pie diagram. Rho GTPase subfamilies and individual Rho GTPases are color coded. (C) The 2^−ΔΔCt^ values of the Rho GTPases in macrophages and osteoclasts. The 2^−ΔΔCt^ values of the individual data points for each cell type are depicted. Donormix 1 and 2 are derived from 9 and 3 donors, resp. Donormix CD14 is derived from the same donors as donormix 2, but monocytes were obtained by elutriation followed by CD14 MACS isolation and differentiated to macrophages.

In the context of immunity and inflammation, macrophage populations express polarized functions depending on their environment. Polarized macrophages are often referred to as M1 (classical activation) and M2 (alternative activation) cells [24]–[25]. For human monocytes, GM-CSF treatment leads to the formation of MΦ-1 macrophages, with features of M1 cells, while the equivalent population following culture in M-CSF has been termed MΦ-2 macrophages with features of M2 cells [26]–[27]. In GM-CSF-differentiated macrophages the levels of Rac2 are highest, while in M-CSF-differentiated macrophages RhoA is the most abundantly expressed Rho GTPase (Figure 7A). Osteoclasts were differentiated directly from monocytes (‘osteoclasts (mono)’) or from monocyte-derived immature DCs (‘osteoclasts (DC)’). In osteoclasts (mono), RhoA is the Rho GTPase with the highest expression, while in osteoclasts (DC) this is Rac2 (Figure 7B). The expression patterns of the Rho GTPases are very similar in the macrophages and osteoclasts (Figure 7C). The 2^−ΔΔCt^ values are generally a bit higher in macrophages differentiated with GM-CSF than in macrophages generated with M-CSF. The osteoclasts derived from monocytes display slightly higher 2^−ΔΔCt^ values than the DC-derived osteoclasts. The main difference between the differently generated macrophages or osteoclasts is the levels of Rac2 and RhoA. Comparing the expression between macrophages and osteoclasts, the expression pattern of the Rho GTPases in osteoclasts (mono) closely resembles that of macrophages differentiated with M-CSF (Figure 7A and B left panels and 7C), with RhoA being the most prominent Rho GTPase. In addition, the expression pattern in osteoclasts (DC) is very similar to that of macrophages differentiated with GM-CSF (Figure 7A and B right panels and 7C), with Rac2 being the most prominent Rho GTPase. Osteoclasts are differentiated with RANKL and M-CSF, but the osteoclasts (DC) are first stimulated with IL-4 and GM-CSF to obtain DCs. These findings show that GM-CSF and M-CSF induce a distinct pattern of Rho GTPase expression, i.e. GM-CSF treatment results in high levels of RhoA and M-CSF treated cells mainly express Rac2 mRNA. In addition, these data suggest that the relatively high RhoA expression, once induced by GM-CSF, is not changed by subsequent stimulation with M-CSF.

## Discussion

Rho GTPases are critical regulators of several aspects of cell behavior, including adhesion and migration. The activity of these GTPases is regulated in a complex manner by GEFs, GAPs and RhoGDI [2]–[4]. However, additional regulatory mechanisms are involved, such as ubiquitylation and degradation [28]. But GTPases are also regulated on the transcriptional level. The family of Rho GTPases contains 20 members, however only a few, i.e. Cdc42, Rac1 and RhoA have been studied in detail.

In myeloid cells, proper regulation of adhesive and migratory behavior is essential. Surprisingly little is known about the expression of Rho GTPases in various types of myeloid cells. To fill this gap, we analyzed the expression of the Rho GTPase family members and splice variants in different human myeloid cells by qPCR. This study is intended to indicate which of the Rho GTPases are expressed in a particular cell type and therefore should be considered when studying functions of these cells that are controlled by Rho GTPases. We chose to investigate mRNA levels instead of protein levels, because there are no good or specific antibodies available for many of the Rho GTPases. The mRNA levels do not necessarily correlate well to protein levels. However, the protein levels we investigated as a proof of principle correlate well with the mRNA levels. We found a general trend showing that Cdc42 splice variant 1, RhoQ, Rac1, Rac2, RhoA and RhoC are the main Rho GTPases mRNAs in myeloid cells. Interestingly, these six Rho GTPases are part of three subfamilies with two Rho GTPases from within each subfamily, i.e.Cdc42 and RhoQ from the Cdc42-subfamily, Rac1 and Rac2 from the Rac-subfamily and RhoA and RhoC from the Rho-subfamily. Therefore, it would be interesting to study the function of these Rho GTPases, alone or in combination, in myeloid cell biology.

**Figure 8:**
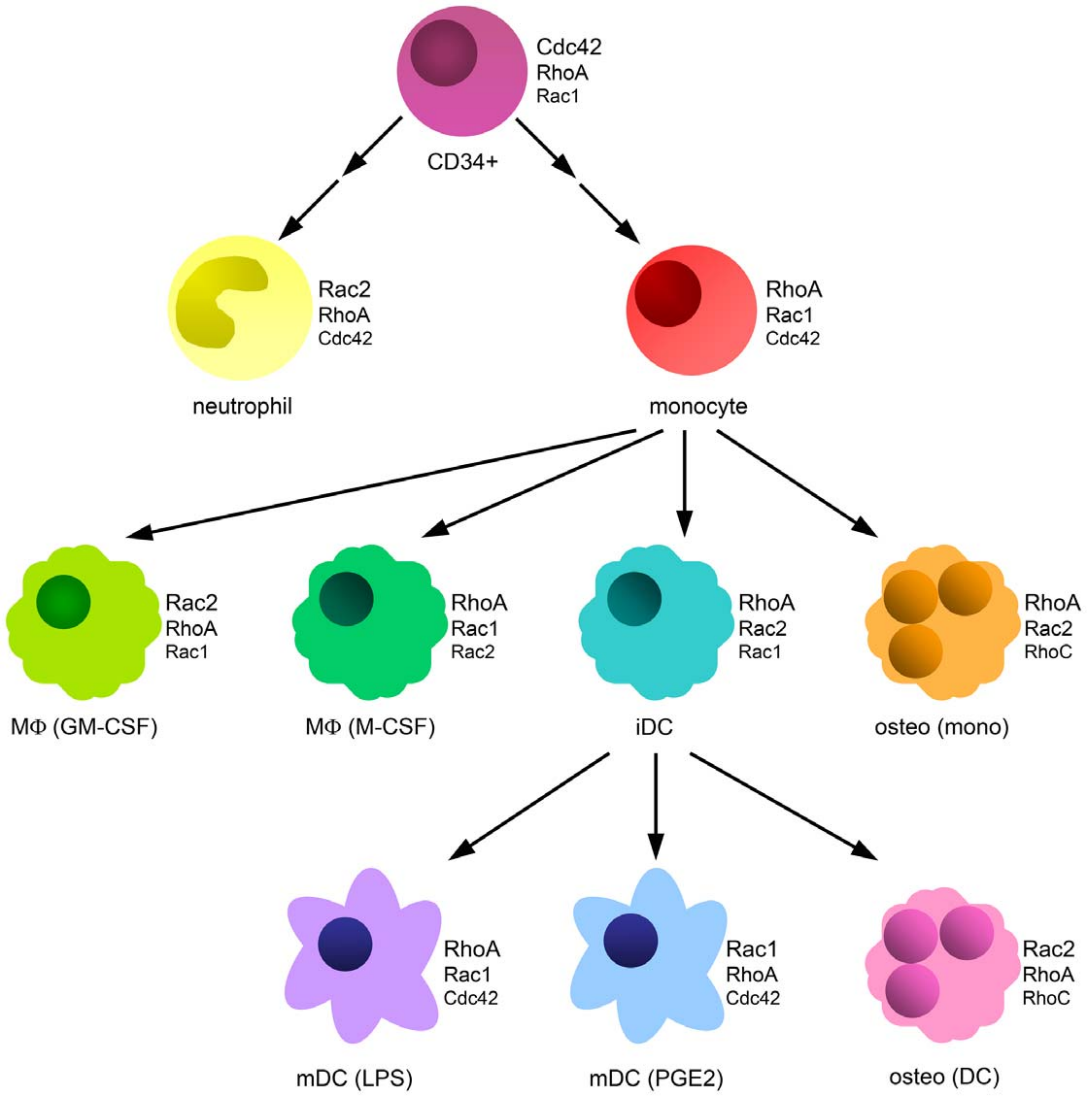
Overview of most abundant Rho GTPases per cell type. The different cells investigated in this study are depicted with the three most highly expressed Rho GTPases next to them. CD34+; CD34^+^ cell, MΦ (GM-CSF); macrophage differentiated with GM-CSF, MΦ (M-CSF); macrophage differentiated with M-CSF, iDC; immature DC, osteo (mono); osteoclast differentiated from monocyte, mDC (LPS); LPS-matured DC, mDC (PGE2); PGE_2_-matured DC, osteo (DC); osteoclast differentiated from DC. An arrow indicates that a cell type is differentiated from the cell type at the start of the arrow. Two arrows between CD34^+^ and monocyte or neutrophil indicate that the monocytes and neutrophils are not directly differentiated from CD34^+^ cells, but that there are intermediate cells in between.

To a lesser extent and depending on the type of cell RhoB, RhoG, RhoF and RhoV are also expressed at mRNA level and have to be taken into account. Since RhoB is an early response gene [29] and is upregulated quickly in response to some stimuli, RhoB could have a prominent function in specific situations where stimulation with, for instance, cytokines increases the mRNA levels of RhoB. The mRNA levels of Cdc42 splice variant 1, RhoJ, the Rac1 splice variant Rac1B, Rac3, RhoD, RhoH, RhoU, the Rnd subfamily and the RhoBTB subfamily are very low or not detected. We found that the relative expression levels of the prominently expressed Rho GTPases varied in different myeloid cells and between progenitor and differentiated cells, confirming that regulation at the mRNA level is important in these cells. An overview of the analyzed cells and the three most highly expressed Rho GTPase mRNAs in each is shown in Figure 8.

Two Rho GTPases have been characterized as restricted to hematopoietic cells, i.e. Rac2 and RhoH [5], [30]. Indeed, we found that Rac2 was prominently expressed in all myeloid cell types analyzed. Low levels of Rac2 were detected in HeLa and neuroblastoma cells, indicating that it is not exclusively expressed in hematopoietic cells. In contrast, expression of RhoH was completely absent from these control cells, confirming the hematopoietic cell-restricted expression profile. The expression of RhoH was low or very low in myeloid cells. RhoH has been suggested to be mainly expressed in the lymphoid lineage and is important for thymocyte selection and T-cell receptor signaling [31]–[32]. Although the levels of RhoH are higher in PBLs than in myeloid cells, these levels are relatively low. RhoH is an atypical Rho GTPase, meaning that it resides mostly in its GTP-bound form [5]. The level of expression of atypical Rho GTPases is lower than that of classical Rho GTPases. From the atypical Rho GTPases (RhoH, RhoU, RhoV, Rnd1-3 and RhoBTB1-2), RhoV was most prominently expressed in myeloid cells, suggesting that this GTPase has an important function in these cells. Moreover, the expression of RhoV is high in CD34^+^ cells and decreases during the differentiation of monocytes towards macrophages, DCs or osteoclasts. These findings suggest that RhoV might be important in controlling the differentiation of myeloid cells, especially along the monocyte-lineage.

Previously, another atypical Rho GTPase, RhoU, was found to be upregulated during murine osteoclast differentiation [33]. Although the overall expression pattern we observed in osteoclasts is similar to the pattern found in this study [33], we observed no or very low expression of RhoU in osteoclasts. This could reflect differences in the experimental set-up or expression differences between humans and mice.

In addition to RhoV, Cdc42 (splice variant 1) also displays a expression profile with somewhat higher mRNA levels in CD34^+^ cells than in differentiated cells of the monocyte-lineage. However, the highest mRNA levels for Cdc42 are found in neutrophils. Cdc42 has been shown to be an important regulator of cell division [34], but is also critical for the formation of podosomes and thereby regulates adhesion [35]–[36]. In addition to Cdc42, also Rac1, Rac2 and RhoA are found at very high levels in neutrophils. This fits well with the expression on protein level for Cdc42, Rac1 and RhoA (our data) and Rac2 [37]. Neutrophils are among the first cells to respond to infection and migrate quickly to the site of infection. Therefore, the high levels of these Rho GTPases in neutrophils could reflect a high demand for Rho GTPase activity which might be required for this fast response.

An additional Rho GTPase that showed a clear expression trend comparing different myeloid cells is RhoC. In contrast to RhoV, RhoC expression is low in CD34^+^ cells and increases during the differentiation of monocytes to the different monocyte-derived cells. The more differentiated cells are more adhesive than precursors or monocytes and make prominent podosomes. Interestingly, RhoC was recently described to regulate invadopodia in cancer cells [38], which are closely related to podosomes in myeloid cells. Therefore, RhoC might be involved in the regulation of podosome formation and in adhesion of myeloid cells. In addition to RhoC, RhoA expression is lowest in CD34^+^ cells, suggesting that these two Rho-subfamily GTPases might be important regulators of myeloid cell differentiation, adhesion and migration.

DCs are distributed throughout tissues, but upon stimulation acquire the ability to migrate to the lymph nodes to trigger the adaptive immune response [1]. Rac1 and Rac2 seem to be regulated differently during DC maturation. The mRNA levels of several Rho GTPases, including Rac1, increase during maturation, while Rac2 levels decrease. This suggests that these two closely related GTPases activate different effector proteins and thereby regulate different aspects of cell behavior. Previously, it was shown by using Rac1/2 single or double knock out mice that Rac2 or Rac1/2 deficient macrophages lack podosomes, while macrophages lacking Rac1 have impaired podosomes [39]. Interestingly, immature DC display relatively high level of Rac2 mRNA and form prominent podosomes. During maturation podosomes are dissolved correlating well with the decrease in Rac2 levels. Furthermore, the same study showed that macrophages deficient for Rac1, Rac1/2, but not Rac2, display impaired matrigel invasion [39]. This also fits well with our findings, since Rac1 levels increase during maturation and DCs gain migratory capacity during this process. Together these findings show that Rac1 and Rac2 have distinct functions in the regulation of myeloid cell adhesion and migration.

Apart from expression trends in distinct myeloid cells, the stimulation with GM-CSF or M-CSF influences the expression pattern of Rho GTPases. GM-CSF stimulation leads to a high expression of Rac2, while M-CSF stimulation leads to a high RhoA expression in macrophages and osteoclasts. The function of osteoclasts in inflammation is unclear, but many defects in inflammation are also accompanied by defects in bone homeostasis [40]–[43]. Osteoclasts are critical for the control of bone mass density and regulate bone resorption; this process depends on the adhesive capacity to the bone surface and the ability to move across this surface [44]. In DC-derived osteoclasts, the early stimulation with GM-CSF during DC differentiation is not reversed by the subsequent stimulation with M-CSF during osteoclast differentiation, suggesting that the expression profile induced by cytokine stimulation during early differentiation is dominant.

This study shows that Rho GTPases are regulated at the RNA level during myeloid differentiation. Furthermore, our findings identify several Rho GTPases that are likely to be important in regulating specific myeloid cell functions, in particular adhesion and migration.

## Materials and Methods

### Cell isolation and culture

The primary human material was obtained after written informed consent and with approval of the Medical Ethical Committee of the Academic Medical Centre (Amsterdam, The Netherlands), which specifically approved this study. Mobilized peripheral blood samples were harvested from leukapheresis material of G-CSF-treated mantle cell lymphoma patient treated with chemotherapy and G-CSF (2×5 µg/kg/day subcutaneously; Filgrastim, Amgen, CA, USA). These patients are in remission and therefore the CD34^+^ cells are probably normal. The samples were diluted in phosphate-buffered saline (PBS; Fresenius Kobi's Hertogenbosch, The Netherlands) with 2 mM ethylenediamine tetra acetic acid (EDTA) and 0.5% w/v bovine serum albumin (BSA; Sigma-Aldrich, Steinheim, Germany). CD34^+^ cells were obtained by magnetic cell sorting (MACS) (Miltenyi Biotec, Bergisch Gladbach, Germany) reaching purities of 95% or more. Neutrophils were isolated from the heparinized blood of healthy human subjects with a Ficoll-Paque PLUS (GE Healthcare, Uppsala, Sweden) gradient and subsequent lysis of erythrocytes as described [45]. Neutrophil preparations were typically greater than 97% pure, with the contaminating cells being mostly eosinophils. Peripheral Blood Mononuclear Cells (PBMCs) were isolated from buffy coats diluted in PBS with 0.45% citrate with a Ficoll-Paque PLUS (GE Healthcare) gradient. Monocytes were derived from PBMCs either by plastic adherence or elutriation followed by CD14 MACS isolation (according to the instructions of the manufacturer, Miltenyi Biotec). To isolate plastic adherent-monocytes, PBMCs were allowed to adhere in RPMI 1640 medium supplemented with 2% pooled human serum. Non-adhesive cells were removed after 1 hour (PBLs) and the monocytes were cultured in RPMI 1640 medium (Gibco, Invitrogen, Paisley, UK) supplemented with 10% FCS (Life Technologies, Breda, The Netherlands) in the presence of IL-4 (500 U/ml) and GM-CSF (800 U/ml, both Cellgenix, Freiburg, Germany) to generate DCs. Immature DCs were harvested at day 7. DCs were matured with lipopolysaccharide (LPS, 2 µg/ml, Sigma-Aldrich) or prostaglandin E2 (PGE_2_, 10 µg/ml, Sigma-Aldrich) and harvested at day 8. Macrophages were generated using a similar protocol, but in the presence of either GM-CSF (80 U/ml) or M-CSF (50 ng/ml, Peprotech, London, UK) and harvested at day 7. Osteoclasts were differentiated from monocytes in the presence of RANKL (100 ng/ml, Peprotech) and M-CSF (25 ng/ml). Alternatively, osteoclasts were differentiated from dendritic cells in RPMI 1640 medium supplemented with 10% FCS and RANKL (100 ng/ml) and M-CSF (25 ng/ml).

Osteoclasts were harvested at day 7.

### Flow cytometry

Cells (1×10^5^) were incubated with PBS with 5% human serum albumin for 10 minutes at 4°C. After washing with cold PBS the cells were incubated with directly-labeled antibodies (BD bioscience, Franklin Lakes, NJ and R&D systems, Adingdon, UK) for 30 minutes at 4°C. Cells were washed and resuspended in 100 µl PBS with 1% human serum albumin. Fluorescence was measured using a FACS LSRII and analyzed with FlowJo (Tree Star Inc., Ashland, OR) and Diva software (BD bioscience). Expression of MHC class I/II, costimulatory molecules and DC-specific markers on DCs were measured by flow cytometry and the expression of MHC molecules, costimulatory molecules and DC-markers was similar to what was previously described [46].

### Tartrate-resistant acid phosphatase (TRACP) staining

Osteoclasts were stained for TRACP to confirm the differentiation. TRACP staining was performed with an acid phosphatase, leukocyte kit according to the manufacturers (Sigma-Aldrich) instructions.

### Immunofluorescence staining

Coverslips were coated with fibronectin (20 µg/ml, Sigma-Aldrich) in PBS for 1 hour at 37°C. Cells were seeded on fibronectin-coated coverslips, left to adhere for 1 to 12 hours and fixed in 3.7% (w/v) formaldehyde (Sigma-Aldrich) in PBS for 10 minutes. Cells were permeabilized in 0.1% (v/v) Triton X-100 (Sigma-Aldrich) in PBS for 5 minutes and blocked with 2% (w/v) BSA in PBS. The cells were incubated with anti-vinculin antibody (hVIN1, Sigma-Aldrich) for 1 hour. Subsequently the cells were incubated with Alexa Fluor 488-labeled secondary antibodies, Texas Red-conjugated Phalloidin (to detect actin, Molecular Probes, Invitrogen) and Hoechst33258 (Molecular Probes) for 45 minutes. Coverslips were embedded in mowiol. Images were obtained by confocal microscopy using a Zeiss LSM 510-meta microscope with a Plan-Apochromatic 63× 1.4 NA oil immersion objective (Carl Zeiss, Jena, Germany) and analyzed using Zen software (Carl Zeiss).

### SDS-PAGE and western blotting

Samples were prepared by directly taken up the cells in sample buffer. Proteins were separated by SDS-PAGE (on 12.5% polyacrylamide gels) and transferred onto nitrocellulose transfer membrane (GE Healthcare). Following blocking in 5% low-fat milk in TBST (tris-buffered saline Tween-20) the blots were incubated with the primary antibody overnight at 4°C. Antibodies against tubulin (Sigma-Aldrich), ERK and RhoA (Santa Cruz Biotechnology, Santa Cruz, CA, USA), RhoC (Cell Signaling Technology, Danvers, MA, USA), Cdc42 and Rac1 (Transduction laboratories, BD bioscience) were used. Next, the blots were washed three times for 10 minutes in TBST and subsequently incubated with HRP-coupled secondary antibodies (dilution 1∶5000, Dako, Glostrup, Denmark) in TBST for 1 hour at room temperature. Finally, blots were washed three times with TBST for 20 minutes each and subsequently developed by ECL (GE Healthcare)

### RNA extraction, Reverse Transcription and qPCR

Total cellular RNA from different types of myeloid cells was extracted with Trizol (Invitrogen) according to the manufacturer's instructions. cDNA was synthesized using 1 µg of total RNA according to the European Against Cancer (EAC) guidelines [47] using Superscript III reverse transcriptase (Invitrogen). Primers for all 22 Rho GTPases and splice variants were designed using Primer Express 1.5 (Applied Biosystems, Foster City, CA) and Oligo 6 (Molecular Biology Insights Inc, Cascade, CO) on the basis of published gene sequences (http://www.ncbi.nlm.nih.gov/). Amplicons span an intron of at least 500 base pairs, except for RhoB that consists of only one exon. The primers for Cdc42 splice variant 1 (Cdc42v1) are specific for Cdc42 transcript variant 1 and 3, which generate the same protein, and the primers for Cdc42 splice variant 2 (Cdc42v2) are specific for transcript variant 2. The primers for Rac1 are specific for both Rac1 and Rac1B, while the Rac1B primers only recognize Rac1B. There are 3 transcript variants of RhoC that differ only in the 5′UTR and thus generate the same protein. The primers used here recognize all 3 variants. Primers were synthesized by Eurogentec (Liege, Belgium).

qPCR (quantitative real-time PCR) was performed in an Applied Biosystems 7900HT Fast Real-Time PCR System (Applied Biosystems, Foster city, CA). Reactions were carried out in 25 µl containing 12.5 µl SYBR GREEN PCR Master Mix (Applied Biosystems), 300 nM forward and reverse primer, 5 µl cDNA (100 ng RNA equivalents) and started with 10 minutes at 95°C followed by 50 cycles of 15 seconds at 95°C and 60 seconds at 60°C. The specificity of all PCR products was determined by melt curve analysis. To correct for differences in the amount of total RNA input and for RT-efficiency, the quantity of the Rho GTPase transcripts was normalized to the amount of β-glucuronidase (GUS) gene transcripts [47]. Primer combinations for GUS and the Rho GTPases are listed in Table 2. The primers for CathepsinK and NFATc1 were described previously [18] and GUS was again used as reference gene.

**Table 2:** Primer sequences.

Name	Accession	Forward primer 5′-3′	Reversed primer 5′-3′
GUS	M15182	GAAAATATGTGGTTGGAGAGCTCATT	CCGAGTGAAGATCCCCTTTTTA
Cdc42v1	NM_001791	CTGTCAAGTATGTGGAGTGTTCTGC	CTCTTCTTCGGTTCTGGAGGCT
Cdc42v2	NM_044472	GTCAAGTATGTGGAGTGTTCTGCAC	GCACTTCCTTTTGGGTTGAGTTT
RhoJ	NM_020663	CAACGACGCCTTCCCAGA	CTTGCCTCCCACAGTCACAGT
RhoQ	NM_012249	CAAGACTGAATGATATGAAAGAAAAACCTA	AAAGCTGAACATTCCACATAGCAG
Rac1	NM_006908	CCTGATGCAGGCCATCAAG	AGTAGGGATATATTCTCCAGGAAATGC
Rac1B	NM_018890	TGAATCTGGGCTTATGGGATACA	GGTTATATCCTTACCGTACGTTTCTCC
Rac2	NM_002872	CACGATGCAGGCCATCAA	GGCGTTGGTGGTGTAGCTG
Rac3	NM_005052	TGATCTGCTTCTCTCTGGTGAGC	GCCGCACCTCCGGGTA
RhoG	NM_001665	GCCTCGGGGAGGGCA	ACTTGATGCTCTGCATCGTGG
RhoA	NM_001664	GGACTCGGATTCGTTGCCT	CCATCACCAACAATCACCAGTT
RhoB	NM_004040	TATTTAAGGGTGGTGATGGGTGA	ATGCTTGGGCGGGAGTCT
RhoC	NM_175744	GAGCACACCAGGAGAGAGCTG	ATGTCCCGGCCTTCCTCA
RhoD	NM_014578	CTGATGGTCTTCGCCGATG	TGACCATGTACCGCTCAAACAC
RhoF	NM_019034	AGCAAGGAGGTGACCCTGAAC	CCGCAGCCGGTCATAGTC
RhoH	NM_004310	TGATTTCCGGAGTCAGTCATTTTA	CGGCTTCAGTTTCTGATGGATC
RhoU	NM_021205	ATGGGCGGCCCGTG	GGCCTCAGCTTGTCAAATTCA
RhoV	NM_133639	GCTCCGGTGCGCATTGA	CTGAAGCACGCCAGGAAGAC
Rnd1	NM_014470	GCAGGCGCCCATCTCC	CCAGGTAGATTTCTGCACCCA
Rnd2	NM_005440	ATCGACAAGCGCCGCA	GCCGGACATTATCATAGTAAGAGGA
Rnd3	NM_005168	TTTGAAATCGACACACAAAGAATAGAG	GGCGGACATTGTCATAGTAAGGA
RhoBTB1	NM_014836	TCCAGCTGTGAGCAGAGTGTTC	GTAGTCCATGTCAGCGTCCATTT
RhoBTB2	NM_015178	CTGGATGGCTGCCATGTTT	GGTATTCCAGCACGGCCC

Accessions are derived from http://www.ncbi.nlm.nih.gov/.

To verify the different primer pairs for their specificity, they were first tested in cell types known to express the different Rho GTPases (PBLs, HeLa and neuroblastoma cells). Each primer pair generated a specific PCR product resulting in the same melting curve peak for each sample (data not shown). Different donors or mixtures of donors were used. Donormix 1 is a mix from 9 different donors. The PBLs and the monocytes from this mixture were used and the monocytes were further differentiated to the two types of macrophages, osteoclasts and DCs. The DCs were further differentiated into osteoclasts or matured with LPS or PGE_2_ to obtain mature DCs. Donormix 2 is derived from 3 different donors and monocytes from this mix were isolated in two different ways, either by plastic adherence (as in donormix 1) or by elutriation followed by CD14 MACS isolation. The monocytes isolated by plastic-adherence were differentiated again into two types of macrophages, DCs (immature and mature) and osteoclasts (from monocytes or DCs). The CD14+ monocytes were differentiated to the two types of macrophages and immature DCs. The mRNA levels for the Rho GTPases in these monocytes, immature DCs and macrophages were similar when different methods of isolation were used. The additional PBL samples were each obtained from an individual donor as were the neutrophil samples. Each CD34^+^ donormix consists of a mix of 3 mantel cell lymphoma patients in remission.

Each individual qPCR was performed in duplo and Ct values were allowed to differ by no more than 0.7 Ct. Analysis were made by comparing either individual or average 2^−ΔΔCt^ values [48] or by comparing the expression of individual Rho GTPases to the total expression of all Rho GTPases in one cell type (percentage). The percentage of total Rho GTPase expression was determined by adding up all the 2^−ΔΔCt^ values of Rho GTPases in a certain cell type (100%) and dividing the 2^−ΔΔCt^ value of the individual Rho GTPase by this.

## Supporting Information

Figure S1 **Rho GTPase expression in myeloid and control cells.** (A) The percentage of total Rho GTPase expression is depicted for myeloid cells, PBLs, HeLa and neuroblastoma cells in a pie chart. Rho GTPase subfamilies and individual Rho GTPases are color coded. The expression pattern is distinct for the different cell types. (B) The 2^−ΔΔCt^ values of the Rho GTPases in control cells. The 2^−ΔΔCt^ values of the individual data points for PBLs, HeLa and neuroblastoma are depicted. Donormix 1 for PBLs is derived from 9 donors.
(TIF)

Figure S2 **Expression pattern of Rho GTPases in myeloid and control cells.** For the 10 most prominently expressed Rho GTPases, i.e. Cdc42, RhoQ, Rac1, Rac2, RhoG, RhoA, RhoB, RhoC, RhoF and RhoV, the 2^−ΔΔCt^ values of the individual data points for myeloid and control cells are depicted. The control cells, i.e. HeLa, neuroblastoma and PBL, are on the right side of the dashed line. Note the different scales on the Y-axes; 30, 10 or 5. CD34+; CD34^+^ cells, iDCs; immature DCs, mDCs (LPS); LPS-matured DCs, mDCs (PGE2); PGE_2_-matured DCs, MΦ (GM-CSF); GM-CSF-differentiated macrophages, MΦ (M-CSF); M-CSF-differentiated macrophages, osteo (mono); monocyte-derived osteoclasts, osteo (DC); DC-derived osteoclasts.
(TIF)
