# Supplementary figures and images for: Rho GTPase Expression in Human Myeloid Cells
Source: PLoS One. 2012 Aug 16;7(8):e42563. doi: 10.1371/journal.pone.0042563 (PMC3420873; doi:10.1371/journal.pone.0042563)

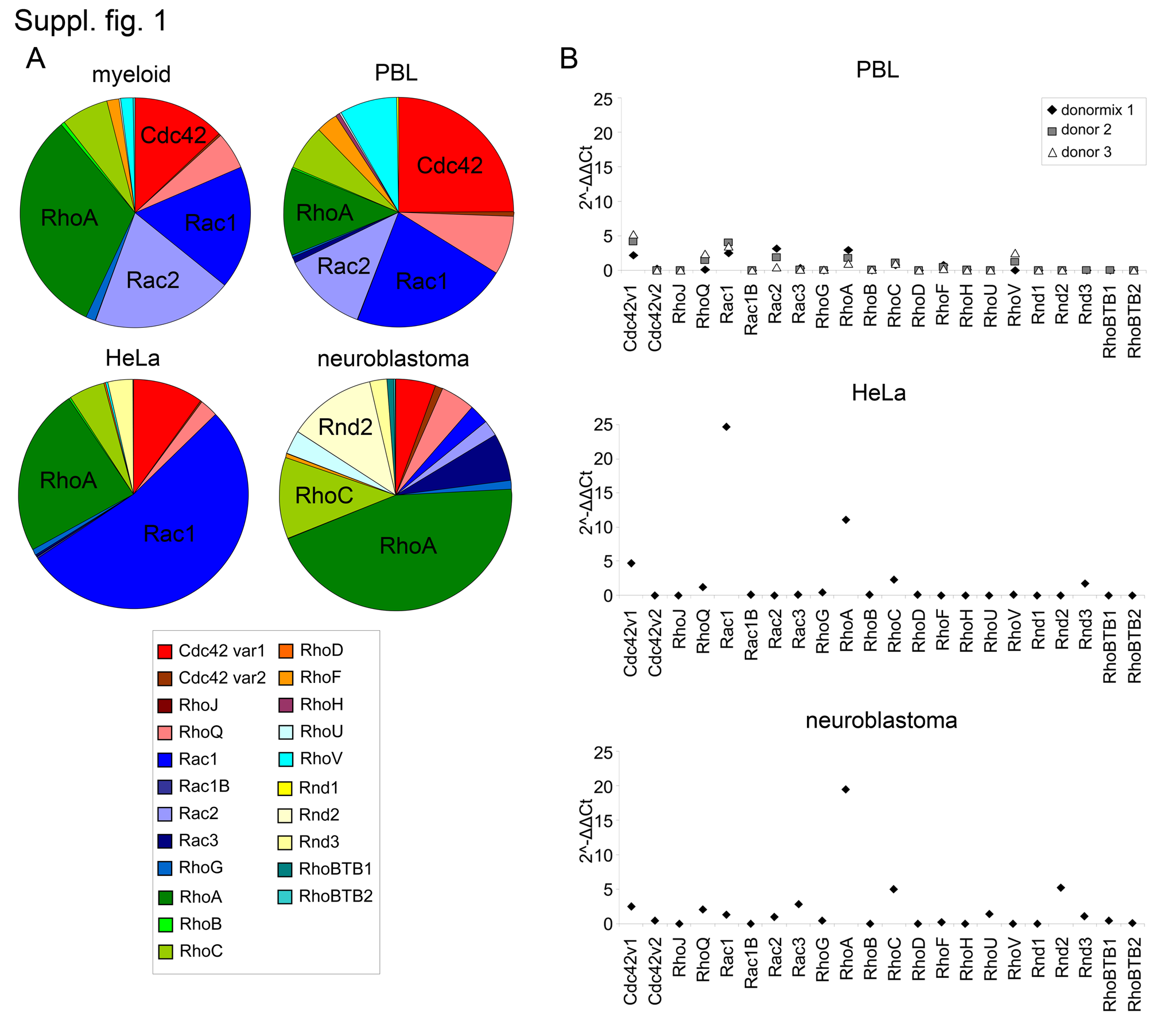

Supplement: Figure S1 — Rho GTPase expression in myeloid and control cells. (A) The percentage of total Rho GTPase expression is depicted for myeloid cells, PBLs, HeLa and neuroblastoma cells in a pie chart. Rho GTPase subfamilies and individual Rho GTPases are color coded. The expression pattern is distinct for the different cell types. (B) The 2−ΔΔCt values of the Rho GTPases in control cells. The 2−ΔΔCt values of the individual data points for PBLs, HeLa and neuroblastoma are depicted. Donormix 1 for PBLs is derived from 9 donors. (TIF) [file pone.0042563.s001.tif]

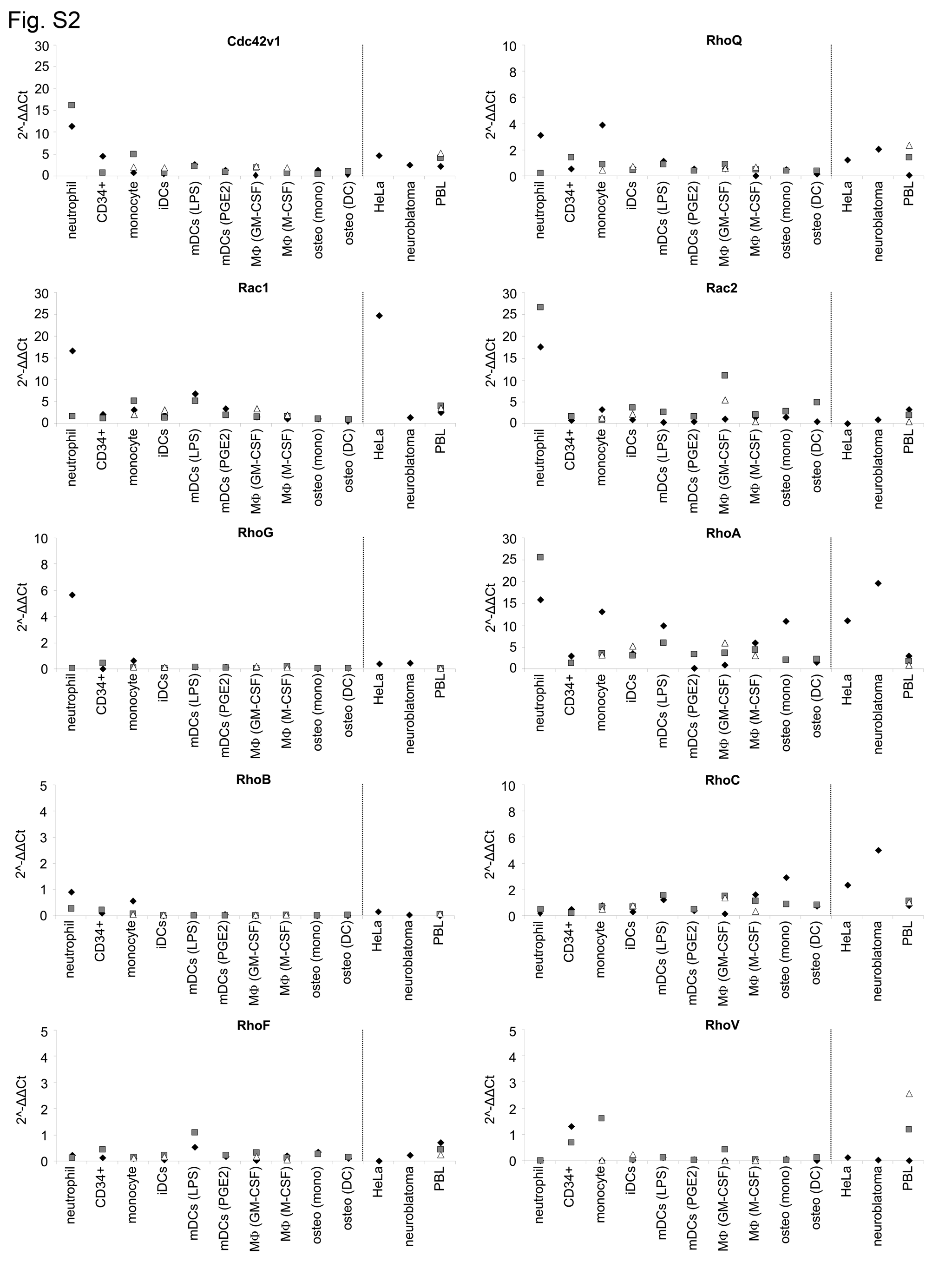

Supplement: Figure S2 — Expression pattern of Rho GTPases in myeloid and control cells. For the 10 most prominently expressed Rho GTPases, i.e. Cdc42, RhoQ, Rac1, Rac2, RhoG, RhoA, RhoB, RhoC, RhoF and RhoV, the 2−ΔΔCt values of the individual data points for myeloid and control cells are depicted. The control cells, i.e. HeLa, neuroblastoma and PBL, are on the right side of the dashed line. Note the different scales on the Y-axes; 30, 10 or 5. CD34+; CD34+ cells, iDCs; immature DCs, mDCs (LPS); LPS-matured DCs, mDCs (PGE2); PGE2-matured DCs, MΦ (GM-CSF); GM-CSF-differentiated macrophages, MΦ (M-CSF); M-CSF-differentiated macrophages, osteo (mono); monocyte-derived osteoclasts, osteo (DC); DC-derived osteoclasts. (TIF) [file pone.0042563.s002.tif]
